# Anti-inflammatory, Antinociceptive, and Toxicological Properties of *Uvaria comperei* Stem Crude Extract and Fractions

**DOI:** 10.1155/2023/2754725

**Published:** 2023-01-23

**Authors:** Marguerite Kamdem Simo, Gael Tchokomeni Siwe, Maurice Taboula Kayo, Zheng Chen, Mersimine Mangoua Kouamo, Darline Dize, Pierre Dongmo Jazet, Modeste Lambert Sameza, Fabrice Boyom Fekam, Guglielmina Froldi

**Affiliations:** ^1^Department of Biochemistry, Faculty of Sciences, University of Yaoundé I, Box 812, Yaoundé, Cameroon; ^2^Department of Biological Sciences, Faculty of Sciences, University of Maroua, Box 814, Maroua, Cameroon; ^3^Department of Animal Biology and Physiology, Faculty of Sciences, University of Yaoundé I, Box 812, Yaoundé, Cameroon; ^4^Department of Chemistry, Higher Teacher Training College, University of Bamenda, P.O. Box 39, Bambili, Cameroon; ^5^Department of Pharmaceutical and Pharmacological Sciences, University of Padova, 35131 Padova, Italy; ^6^Department of Biochemistry, Faculty of Sciences, University of Douala, Box 24157, Douala, Cameroon

## Abstract

The present study was carried out to investigate the anti-inflammatory activity of a methanolic extract and fractions of *Uvaria comperei* stems. The crude extract was obtained by maceration of the powder in methanol and fractions by vacuum chromatography from the methanolic extract. To study the anti-inflammatory activity *in vitro*, red blood cell lysis inhibition assay and albumin denaturation inhibition were performed, while *in vivo* measurements of carrageenan-induced paw oedema and formalin-induced pain in albino mice were performed. Acute toxicity and cytotoxicity studies of the fraction F2 were performed, as well as its HPLC, and some biochemical parameters were quantified. *Uvaria comperei* crude extract (UCCE) at 250 and 500 *μ*g/mL completely inhibited albumin denaturation, while decreasing 75.5% of heat blood cell lysis at 500 *μ*g/mL. The fractions 128-136 (F3), 10-11 (F1), and 56-62 (F2) at 500 *μ*g/mL displayed a significant anti-inflammatory activity with percentages of inhibition of 60.5, 67.4, and 100%, respectively. Administration of fraction F2 (25, 50, and 100 mg/kg, p.o.) produced a dose-dependent inhibition of formalin-induced pain of 60.2% at 50 mg/kg in the neurogenic phase (*p* < 0.05) and 70.2% at 25 mg/kg in the inflammatory phase (*p* < 0.05). Similarly, the time-dependent increase in carrageenan-induced paw circumference induced by carrageenan was inhibited by pretreatment with F2: 50% of inhibition at 25 mg/kg after 30 min (*p* < 0.05) and 96.5% inhibition at 25 mg/kg after 6 h (*p* < 0.05). In this research, the fraction F2 presented its maximum analgesic property at 50 mg/kg, while it presented the highest anti-inflammatory property at 25 mg/kg. The oral lethal median dose (LD_50_) of F2 was determined to be greater than 2000 mg/kg; further low cytotoxicity in RAW cells was also observed. Overall, this work shows that the methanolic crude extract and fractions, mainly F2, of *Uvaria comperei* stem have interesting anti-inflammatory properties.

## 1. Introduction

Several species belonging to the Annonaceae family are widely known and used in folk medicine and commercial manufacturing of phytotherapeutic products. Among them, plants of the genus *Uvaria* are traditionally used for the treatment of dysentery, wounds, abdominal ache, and malaria [1]. However, very few ethnobotanical and pharmacological studies have been conducted on *Uvaria comperei* species despite their good bioactivity properties [2, 3].

*Uvaria comperei* is a liana with a blackish bark. Their leaves are long-stalked and long, wide limbs rounded at the base. The seeds, wide, are biseriate, in the shape of a flattened ellipsoid and with a brown testa, finely honey-combed [4]. *Uvaria comperei* contains a wide variety of secondary metabolites, mainly phenolic compounds [2, 3].

Phenolic compounds are a well-known group of secondary metabolites with various pharmacological activities [5]. According to Loomis and Battaile [6], phenols belong to either one of the two biochemical groups: flavonoid compounds (including condensed tannins) and the group of compounds where the 6-carbon ring has a 1 or 3 carbon side chains and its derivatives, e.g., caffeic acid, gallic acid, hydrolysable tannins, and lignin. Flavonoids and phenolic acids are the important secondary metabolites and bioactive compounds in plants [7]. Flavonoids are an important coloring component of flowering plants and are found in several plant-based foods [8]. In nutrients, flavonoids are generally responsible for color, taste, prevention of fat oxidation, and protection of vitamins and enzymes. Furthermore, flavonoids are important for human health due to their pharmacological activities as radical scavengers [9]. Several epidemiological studies also suggested protective effects against cardiovascular diseases, cancer, and other age-related diseases [9]. Some flavonoids have been reported to have a variety of biological activities, including antiallergic, antiviral, antiproliferative, anticarcinogenic, and anti-inflammatory activities [10].

Inflammation is a complex biological response in which vascular tissues respond to harmful stimuli such as irritants, pathogens, and damaged cells [11]. Inflammation is commonly divided into three phases: acute inflammation, immune system response, and finally chronic inflammation [12–14]. The inflammation response implicates macrophages and neutrophils that secrete a number of mediators (eicosanoids, oxidants, cytokines, and lytic enzymes) responsible for the initiation, progression, and persistence of the acute or chronic state of inflammation [15]. Following the release of these mediators, inflammatory processes cause tissue damage accompanied by pain.

Researchers are still battling to develop more effective and less toxic agents to treat signs and symptoms of acute inflammation, as well as the consequences of chronic inflammatory diseases such as pain. The search for new drugs capable of disrupting the inflammatory process, mainly from natural substances, is an important issue in scientific research. The purpose of this research was to study the antinociceptive and anti-inflammatory potential of extracts and fractions of *Uvaria comperei* stems that are not reported in the literature.

## 2. Materials and Methods

### 2.1. Animals

Swiss mice (20–30 g) and Wistar rats (100–150 g) were randomly housed in appropriate cages at room temperature and subjected to a natural day/night cycle with access to food and water *ad libitum*. Animals were allowed to have a period of acclimatization before experimentation. Groups of six animals were used for each protocol. This study was consistent with the Guide for the Care and Use of Laboratory Animals, published by the US National Institutes of Health (NIH publication 85-23, revised 1996). Authorisation for the use of laboratory animals in this study was obtained from the Cameroon National Ethics Committee (number FWA-IRB00001954).

### 2.2. Plant Collection

*Uvaria comperei* plant was harvested in February 2013 in Mount Kalla in the central region of Cameroon and identified by the botanist Dr. Nana. A voucher specimen of the plant (52882/HNC) has been deposited at the Cameroon National Herbarium in Yaoundé.

### 2.3. Extract Preparation

Fresh stems were chopped, air dried, and ground into powder. Hundred grams of powder was introduced into a conical flask and soaked for three days in 500 mL of methanol at room temperature. The resulting mixture was filtered through a filter paper (Whatman No. 3) and then roto-evaporated until complete alcohol evaporation was obtained. The resulting extract (11.83 g) was conserved until further use.

### 2.4. Fractionation of Methanolic Extract

The stem methanolic extract was fractionated by vacuum chromatography using silica gel 40 (0.2-0.5 mm) and eluted with solvents of increasing polarity, hexane (Hex), ethyl acetate (EtOAc), and methanol (MeOH), leading to several fractions. The elution was done successively with a gradient system of Hex-Hex/EtOAc-EtOAc-EtOAc/MeOH-MeOH (from 100% hexane to 100% methanol). Each elution (400 mL) was evaporated to dryness under reduced pressure, and 202 fractions were obtained and grouped, according to their thin-layer chromatographic profile using silica gel 60 F254 (Merck, USA), to obtain 28 new fractions. UV light (*λ*_max_ = 254 nm and 366 nm) and 50% aqueous sulfuric acid were used to visualize TLC plates. The three fractions (F1, F2, and F3) that had appreciable antioxidant activity [2] were successively tested to investigate their antinociceptive and anti-inflammatory activities.

### 2.5. *In Vitro* Assays

#### 2.5.1. Inhibition of Albumin Denaturation

The method of Vidhu et al. [16] and Sangita et al. [17] was used for the denaturation protein assay. A solution of BSA (10 mg/mL) was prepared in phosphate buffer at pH 7.4. Stock solutions of crude extract, fractions, and diclofenac (the reference standard) were prepared at 1 mg/mL. From these stock solutions, five different concentrations (31.25, 62.50, 125, 250, and 500 *μ*g/mL) were obtained. A volume of 0.2 mL of BSA was transferred to Eppendorf tubes, then 2 mL of extract or standard at different concentrations was added, and then 2.8 mL of PBS was added for a total volume of 5 mL. Controls were prepared without extracts (instead of 2 mL of extract, 2 mL of PBS was added). The solutions were incubated at 37°C for 30 min and heated at 70°C for 15 min. The absorbance (Abs) was determined at 660 nm. The percentage of inhibition of protein denaturation was determined on a percentage basis relative to the control, using the following formula:

(1) $$\begin{array}{c} \text{Percentage inhibition }\left(\%\right)=\frac{\left(\text{Abs of control}-\text{Abs of test}\right)\times 100}{\text{Abs of control}} \end{array}$$

#### 2.5.2. Antihaemolytic Activity

*(1) Red Blood Cell Suspension*. The method of Azeen et al. [18] was used for red blood cell lysis with some modifications. Rat blood was obtained by puncture, collected in heparinized tubes, and centrifuged at 3000 rpm for 15 min. Then, plasma was removed, and red blood cells (RBCs) were washed three successive times using saline solution.

*(2) Heat-Induced Haemolysis*. Stock solutions of crude extract, fractions, and standards (diclofenac and ibuprofen) were prepared at 1 mg/mL. From these stock solutions, five different concentrations (31.25, 62.50, 125, 250, and 500 *μ*g/mL) were obtained. In each test tube, 500 *μ*L of NaCl was added consecutively with 500 *μ*L of extract or each fraction, 500 *μ*L of buffer solution, and finally 500 *μ*L of RBC suspension. The test tubes were homogenized. The reaction mixture was incubated in a 56°C water bath for 30 min. After incubation, the tubes were cooled under running tap water and then centrifuged at 3000 rpm for 10 min, and the absorbance of the supernatants was assessed at 560 nm. The controls were prepared without extract or fractions. The percentage of protection against heat-induced haemolysis was calculated using the following formula:

(2) $$\begin{array}{c} \text{Percentage inhibition }\left(\%\right)=\frac{\left(\text{Abs of control}-\text{Abs of test}\right)\times 100}{\text{Abs of control}} \end{array}$$

Based on these data, only the fraction F2 that had the highest activity was selected for *in vivo* studies.

### 2.6. *In Vivo* Assays

#### 2.6.1. Formalin Assay

To assess the antinociceptive effect, the method described by Hunskaar and Hole [19] was used. A volume of 20 *μ*L of 1% formalin solution was injected into the subplantar left hind paw of mice. Mice were observed, and the amount of time (seconds) spent licking and biting the injected paw was measured as an indicator of pain. Responses were measured for 5 min after formalin injection (first phase and neurogenic phase) and 15–30 min after formalin injection (second phase and inflammatory phase). Treatments with saline (p.o.), fraction F2 (25, 50, and 100 mg/kg, p.o.), and indomethacin (10 mg/kg, p.o.) were administered 30 min before formalin injection (*n* = 6 for each group). The percentage of antinociceptive activity was determined using the following formula [20]:

(3) $$\begin{array}{c} \text{PAA}=\frac{\text{Tn}-\text{Tt}}{\text{Tn}}\times 100, \end{array}$$

where PAA is the percentage of antinociceptive activity, Tn is the licking time of the control group, and Tt is the licking time of each tested group.

#### 2.6.2. Carrageenan-Induced Hind Rat Paw Oedema Assay

Anti-inflammatory activity was studied using the carrageenan-induced paw oedema model induced by a 1% carrageenan solution, injected at a volume of 100 *μ*L/animal into the subplantar region of the right hind paw of the rats [21]. The rats were divided into six groups, each of six animals. Rats were pretreated with fraction F2 (25, 50, and 100 mg/kg, p.o.), saline (p.o.), or indomethacin (10 mg/kg, p.o.) 30 min before carrageenan injection. The volume of the rat pedal was measured at 0.5, 1, 2, 3, 4, 5, and 6 h after carrageenan injection. The inhibition of the paw oedema was calculated using the following formula: *V*_*B*_ − *V*_*A*_/*V*_*A*_, where *V*_*A*_ is the volume of the right hind paw before carrageenan injection and *V*_*B*_ is the volume of the right hind paw after carrageenan injection.

### 2.7. Biochemical Parameter Analysis

After the carrageenan assay, the animals were sacrificed and blood was collected in heparinized tubes and centrifuged at 3000 rpm for 30 min to obtain serum. Alanine aminotransferase (ALT), aspartate aminotransferase (AST), *γ*-glutamyl transpeptidase (*γ*-GT), and catalase (CAT), as well as reduced glutathione (GSH) and reactive protein (CRP), were determined using commercial assay kits (Randox, UK; Vitros, USA) according to the manufacturer's protocol.

### 2.8. Cytotoxicity Assay

#### 2.8.1. Cell Culture

African green monkey kidney cells (Vero) and macrophage (RAW 264.7) cells were cultured in DMEM containing 10% fetal bovine serum (FBS) and 1% penicillin-streptomycin. Complete DMEM (500 *μ*L) was prepared with 50 *μ*L of FBS, 5 *μ*L of antibiotics, and 445 *μ*L of simple DMEM.

#### 2.8.2. Resazurin Reduction Assay

The cytotoxicity study was performed by a resazurin reduction assay on Vero and RAW cell lines according to the protocol of Kuete et al. [22] and O'Brien et al. [23]. This assay is based on the reduction of the indicator dye, resazurin, to highly fluorescent resorufin by viable cells. Nonviable cells rapidly lose metabolic capacity to reduce resazurin and thus produce no fluorescent signal. Briefly, adherent cells were removed by trypsin treatment, incubated at 37°C for 5 min. Then, trypsin was deactivated by adding complete DMEM, and the solution was centrifuged. An aliquot of 10,000 cells was placed in each well of a 96-well cell culture plate (100 *μ*L). The microplates were incubated at 37°C overnight. After incubation, the medium was removed from each well, and 90 *μ*L of fresh, complete DMEM and 10 *μ*L of fraction solution were added. The plates were then incubated at 37°C for 44 h. Fluorescence was measured on an Infinite M2000 Pro™ plate reader (Tecan, Germany) using an excitation wavelength of 530 nm and an emission wavelength of 590 nm. Each assay was performed at least three times, with six replicates each. Viability was evaluated by comparison with untreated cells. IC_50_ values represent the concentration required to inhibit 50% of cell proliferation.

### 2.9. Acute Toxicity Study

Estimation of the oral median lethal dose (LD_50_) of the fraction was determined in female mice using the OECD Guideline 425 [24]. A limit toxicity test of a single dose of 2000 mg/kg was used. Four hours before the toxicity tests, the animals were deprived of food, with unrestricted access to water. After weighing the mice, three groups of three mice were constituted as follows: group 1 control lot received only 5% DMSO, group 2 mice received the fraction F2 from *Uvaria comperei* extract at 2000 mg/kg, and group 3 mice received only food and water. After oral administration of different substances, mice were monitored and individually observed every 30 minutes, during the day, and then daily for 14 days.

### 2.10. High-Performance Liquid Chromatography with Diode Array Detection Analysis

High-performance liquid chromatography (HPLC) analysis was performed using a HPLC system consisting of a Waters 1525 binary HPLC pump and a Waters 2998 photodiode array detector. Separation was carried out on a RP 18 column (4.6 mm × 15 mm × 3.5 *μ*m) (Waters, USA). Gradient elution was performed at 25°C with solution A (water and 1% acetic acid) and solution B (methanol and 5% acetic acid) in the following gradient from 0% to 100% solution B in 50 min. The flow rate was 1 mL/min, and the injection volume was 20 *μ*L. The peaks were detected at the wavelengths of 240, 340, and 380 nm. Before injection, each sample (1 mg/mL) was filtered through a 0.45 *μ*m membrane filter. The identification of compounds was performed on the basis of retention time, coinjection, and spectral matching with standard compounds. For this purpose, standard stock solutions of caffeic acid, catechin, chlorogenic acid, gallic acid, herniarin, imperatorin, and quercetin were prepared in methanol at 1 mg/mL.

### 2.11. Statistical Analysis

All data are expressed as mean ± SEM and analyzed with SPSS 22.0 software using one-way analysis of variance (ANOVA) followed by Fisher's LSD test. Values of *p* < 0.05 were considered statistically significant.

## 3. Results

### 3.1. *In Vitro* Assays

#### 3.1.1. Inhibition of Albumin Denaturation

The methanolic extract of *Uvaria comperei* and fractions showed a strong inhibitory effect on the heat-induced denaturation of albumin (Table 1). The maximum effect was presented by the crude extract (UCCE) and the F2 fraction employed at 500 *μ*g/mL, obtaining the maximal inhibition, whereas at 500 *μ*g/mL, diclofenac showed an inhibition of 46%. The crude extract showed the highest inhibition (IC_50_ = 66.05 ± 0.30 *μ*g/mL, Table 1).

#### 3.1.2. Heat-Induced Haemolysis

The protective effect of the extract and fractions against heat-induced haemolysis was studied showing a concentration-dependent inhibition (Table 2). In fact, the haemolysis ratio gradually decreased with increasing amount of the substances. Protection was slightly manifested using 31.25 *μ*g/mL crude extract and inhibition of 10.5%, while maximum protection of 95.4% was observed using 500 *μ*g/mL F3 fraction, followed by F2 fraction (79.8%). F3 showed the highest protection for red blood cells, with an IC_50_ < 31.25 *μ*g/mL (as for ibuprofen).

### 3.2. *In Vivo* Anti-inflammatory Assays

#### 3.2.1. Inhibition of Formalin Assay

Treatment with fraction F2 produced significant antinociceptive activity compared to controls in both the early and late phases (Table 3). F2 fraction tested at 25, 50, and 100 mg/kg decreased the paw licking time to 54.4, 60.2, and 14.6%, respectively, in the neurogenic phase (first phase), as well as to 70.2, 68.7, and 38.8%, respectively, in the inflammatory phase (second phase). Indomethacin exhibited a higher inhibition in the second phase.

#### 3.2.2. Inhibition of Carrageenan-Induced Hind Paw Oedema

Oral administration of the F2 fraction (25, 50, and 100 mg/kg) induced the highest anti-inflammatory activity by reducing the volume of paw oedema induced by carrageenan (Table 4). In detail, 25 mg/kg of F2 caused 50% inhibition after 30 min of inflammatory stimulus and 96% after 6 h, while at 50 mg/kg, the inhibition was 87% after 6 h. Furthermore, a 93% inhibition was observed after 3 h with F2 at 50 mg/kg. No significant differences in the inhibition were observed between 25 and 50 mg/kg doses.

### 3.3. Serum Biochemical Analysis

Several biochemical parameters (Table 5) and indicators of oxidative stress (Table 6) were evaluated, as well as C-reactive protein (CRP). The F2 fraction decreased levels of ALT, AST, and *γ*-GT and increased CAT and GSH levels, compared to the control group (*p* < 0.05).

### 3.4. Cytotoxicity

The F2 fraction showed concentration-dependent cytotoxicity activity tested in two cell lines (Table 7). The IC_50_ values of F2 were 27.73 and 82.86 *μ*g/mL, in RAW and Vero cells, respectively.

### 3.5. Acute Toxicity

The F2 fraction did not induce death of any treated mice; therefore, the lethal dose 50 (LD_50_) of F2 was found to be greater than 2000 mg/kg.

### 3.6. HPLC Profile

**Figure 1:**
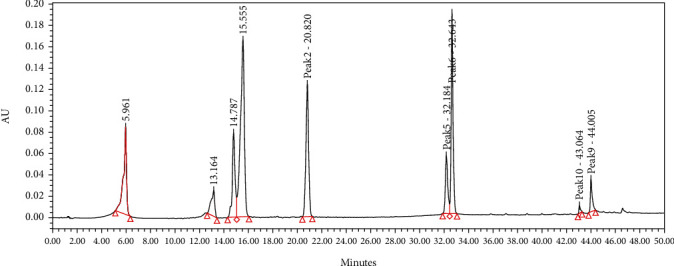
HPLC chromatogram of several standard phenols detected at 280 nm.

**Figure 2:**
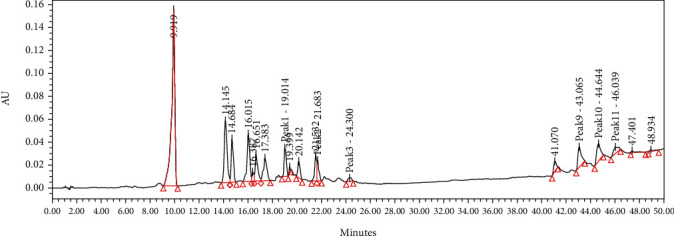
HPLC chromatogram of F2 fraction of *Uvaria comperei* stem extract at 280 nm.

**Figure 3:**
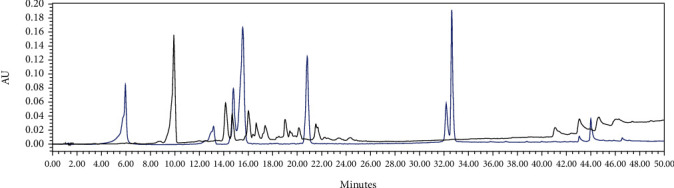
HPLC chromatogram of F2 fraction combined with the standards at 280 nm.

**Figure 4:**
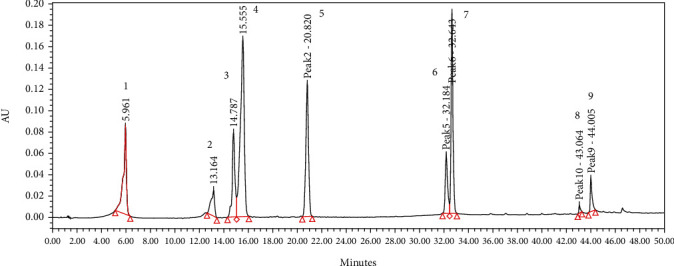
HPLC chromatogram of standards at 280 nm. Peaks: 1, gallic acid; 2, epicatechin; 3, chlorogenic acid; 4, caffeic acid; 5, herniarin; 6, quercetin; 7, imperatorin; 8 and 9, catechin.

Several flavonoids were detected in F2 fraction with HPLC-DAD analysis at 280, 340, and 380 nm and compared with the chromatograms of standard flavonoids.  Figure 1 and Figures S1 and S2 show the HPLC chromatograms of standard phenols (caffeic acid, catechin, chlorogenic acid, epicatechin, gallic acid, herniarin, imperatorin, and quercetin) at 280 nm, 340 nm, and 380 nm, respectively.  Figure 2 and Figures S3 and S4 show the HPLC chromatograms of F2 fraction at 280 nm, 340 nm, and 380 nm, respectively. The chromatograms of the standards combined with F2 fraction have been presented in Figure 3 and Figures S5 and S6, while Figure 4 shows the chromatogram of eight standard flavonoids identified at 280 nm.  Table 8 presents the different phenols identified in fraction F2.

Table 8 shows the flavonoids identified in F2 fraction. The chromatogram revealed that F2 fraction contains numerous phenols; moreover, chlorogenic acid and catechins were identified by the phytochemical analysis.

## 4. Discussion

A previous analysis of the crude stem methanol extract of stems (UCCE) and the F2 fraction of *Uvaria comperei* demonstrated its high antioxidant activity and the characteristic presence of phenols, flavonoids, tannins, and anthraquinones [2, 3]. The high amount of polyphenols in these extracts can explain their scavenging and antioxidant activities. Knowing that radical species are involved in the inflammation process as inductors, previous results suggest the potential anti-inflammatory activity of the UCCE and F2 fraction. Effectively, for the first time, the present research shows the antinociceptive and anti-inflammatory properties of the crude methanol extract of the stem and F2 fraction of *Uvaria comperei* in several *in vitro* and *in vivo* models. In detail, albumin denaturation, heat haemolysis, formalin-induced paw licking, and carrageenan-induced hind paw oedema tests were used to evaluate antinociceptive and anti-inflammatory activities of the UCCE and F2 fraction.

The crude extract and the F2 fraction at 500 mg/mL completely inhibited heat-induced albumin denaturation; furthermore, UCCE showed greater activity than the fractions. It is well known that protein denaturation is a process by which tertiary and secondary structures change, causing loss of protein biological function of proteins. Moreover, one of the main features of inflammation is protein denaturation [25]. In fact, many disorders such as serum disease, rheumatoid arthritis, glomerulonephritis, and systemic lupus erythematosus are the result from hypersensitive reaction, which, in turn, is related to the antigens produced during protein denaturation [26]. Data from the literature suggest that the antidenaturation property of BSA is due to the presence of binding sites in the aromatic tyrosine-rich function and aliphatic regions of the threonine and lysine residues of BSA [27]. According to Verma et al. [28], inhibition of the protein denaturation process by plant-derived extracts can be due to the presence of flavonoids. Several studies have shown that interaction with polyphenolic compounds improved protein thermal stability [5, 29].

The *Uvaria comperei* products effectively inhibited heat-induced haemolysis with a 75% inhibition by using 500 *μ*g/mL UCCE extract, while the 500 *μ*g/mL F1, F2, and F3 fractions exhibited an inhibition of 66, 80, and 95%, respectively. These results suggest that these may inhibit the release of the neutrophil lysosomal content of neutrophils at the inflammation sites. Indeed, the erythrocyte membrane is analogous to the lysosomal membrane, and its stabilization implies that the extract could stabilize the lysosomal membranes, which is important in limiting the inflammatory response by preventing the release of activated neutrophil lysosomal components causing further tissue inflammation and damage. Nonsteroidal drugs act by inhibiting these lysosomal enzymes or stabilizing the lysosomal membrane [30]. The membrane-stabilizing effect of UCCE and F2 could be due to the quality and quantity of phenolic compounds, as UCCE and fraction F2 have a high tenor of polyphenols [2, 3]. Consistent with this idea, Bouhlali et al. [29] showed high correlations between the stabilizing effect of the membrane and phenol contents. The authors suggested that flavonoids may interact at the water-lipid interface with the polar head of phospholipids increasing membrane rigidity, reducing fluidity, and increasing the stability of the mechanical lipid bilayer [31].

The antinociceptive effects of the F2 fraction showed a dose-dependent reduction in pain, which was greater compared to the indomethacin-induced effect. The response to formalin follows a biphasic pattern composed of an initial acute phase (first phase) and then of a longer period (second phase), while the period between phases is called the quiescent interval. Phase I consists of neurogenic nociception by direct stimulation of nociceptors via C fibers to the dorsal horn of the spinal cord after substance P is secreted and acts as a neurotransmitter. The second phase consists of inflammatory-induced pain due to the release of serotonin, histamine, bradykinin, and prostaglandins from formalin-damaged tissue [32]. The pain response in both phases is processed at the spinal level. The spinal cord contains mechanisms that inhibit the activity of neurons that receive and transmit nociceptive information. The primary afferent fibers of the spinal cord utilize excitatory amino acids (EAs) such as glutamate and aspartate as their neurotransmitters. There is evidence that selective EA receptor antagonists produce antinociception [33]. Hence, the antinociceptive activity of the F2 fraction may be due to the capacity to act on the EA receptors or to inhibit phospholipase or cyclooxygenase that participate in the synthesis of prostaglandins. The first phase is reported to be inhibited by opioid analgesics, and the second phase is inhibited by both nonsteroidal anti-inflammatory drugs (NSAIDs) and opioid analgesics. The antinociceptive activity of the F2 fraction could be due to the presence of flavonoids and phenols detected by HPLC analysis and also shown in previous data [2]. However, the cellular mechanism involved in the antinociception of the F2 fraction needs further research, as it was not investigated here.

The F2 fraction had the highest anti-inflammatory activity by reducing the carrageenan-induced paw oedema. It has shown 50% of inhibition just 30 min after carrageenan-induced reaction and 96% after 6 h using 25 mg/kg of F2. Its activity was higher than that of indomethacin, used as standard drug. Previous studies have shown that carrageenan-induced paw oedema is usually a biphasic process. The early stage (0–1 h) is characterised by the secretion of histamine, serotonin, and bradykinin and overproduction of prostaglandins in the surrounding damaged tissue. The later stage (1–6 h) is the target of the most clinically effective anti-inflammatory drugs due to an overproduction of proinflammatory mediators such as bradykinin, leukotrienes, prostaglandins, platelet-activating factor, nitric oxide, and proteolytic enzymes by neutrophils in the inflamed tissues [29]. The present study revealed that the F2 fraction decreased the paw oedema in both phases, different to indomethacin. The low activity of indomethacin in the early stage is expected because nonsteroidal anti-inflammatory drugs such as aspirin or indomethacin are unable to inhibit the early stage of swelling [34]. The inhibition of the paw oedema during the two phases of inflammation suggests that F2 fraction could inhibit various chemical mediators of inflammation. Therefore, it can be speculated that F2 fraction contains phytoconstituents that might be acting through the inhibition of various mediators implicated in the inflammatory damage. Based on the well-known involvement of free radicals in inflammation, it seems that at least a part of the anti-inflammatory effects of F2 fraction may also be attributed to its antioxidant activity [3]. Among the constituents, phenols and flavonoids as chlorogenic acid and catechin were identified in F2 fraction. According to Kimura et al. [35], chlorogenic and caffeic acids also inhibited the histamine and leukotriene production. Other authors reported that luteolin and quercetin inhibited the release of histamine, prostaglandin, and leukotrienes [36], while ferulic and caffeic acids inhibited the enzymes COX-1 and COX-2 [37]. Gallic acid inhibited the production of histamine and proinflammatory cytokines such as TNF-*α* and IL-6 from human-activated mast cells [38].

Biochemical parameters and oxidative stress markers were also evaluated. Administration of fraction F2 in the test group significantly decreased ALT, AST, and *γ*-GT levels compared to the control group. Furthermore, GSH and catalase levels were significantly decreased in the control group compared to the normal group, while the treatment with the F2 fraction (tested group) brings the levels close to the normal group. These observations converge with those of [39], who observed that aqueous and ethanolic leaf and root extracts of *Uvaria chamae* were not significant in rats at the level of uremia and creatinemia, but a sharp increase in the rates of AST and ALT was observed compared to the control. The results suggest that the fraction F2 could have hepatoprotective properties. The decrease in GSH and catalase activity in the control group compared to the normal and test groups suggests that the fraction F2 may have antioxidant activity. This property could be due to the bioactive substances in fraction F2, such as flavonoids, which are the main antioxidant metabolites [5].

The oral median lethal dose (LD_50_) of fraction F2 in mice was found to be greater than 2000 mg/kg body weight. This meant that the extract was practically nontoxic according to the acute toxicity classification standard, thereby validating the ethnomedicinal use of the plant. Reduction in body weight and relative organ weight is generally considered a toxic effect of the extract on the animal, resulting in reduced food and water intake. There was no visible difference between mice in the tested group (G2) and mice in the normal group (G3); however, one death was observed in group 3 on day 14. This death may be incriminated by conditions of storage or physiological parameters of mice. The estimated value of LD_50_ was in agreement with the work of Legba et al. [39] who reported that ethanol and aqueous extracts of the leaves and roots of *Uvaria chamae* were not toxic at 2000 mg/kg in rats. No mortality and no renal histological conditions were recorded in the treated rats.

The fraction F2 showed concentration-dependent cytotoxicity activity against the tested cells. The IC_50_ values shown by F2 were 27.73 and 82.86 *μ*g/mL, respectively, in RAW and Vero cells. According to the American National Cancer Institute (NCI), the cytotoxicity criteria of cytotoxicity for crude extracts were IC_50_ < 30 *μ*g/mL after an exposure time of 72 h in a preliminary assay [40]. Fraction F2 met this criterion with an IC_50_ value less than 30 *μ*g/mL on RAW cells but was slightly cytotoxic in Vero cells.

## 5. Conclusion

This research revealed for the first time that the crude extract and the F2 fraction of *Uvaria comperei* possess high anti-inflammatory activity compared to positive controls, diclofenac and indomethacin. Furthermore, a dose-dependent antinociceptive effect of the F2 fraction has also been observed. *In vivo* hepatoprotective properties could be suggested. Also, the F2 fraction was not toxic up to 2000 mg/kg. The results support the traditional use of *Uvaria comperei* and give credence to the ethnopharmacological approach for the selection of specific plant species for the discovery of new anti-inflammatory agents from natural sources.

## Figures and Tables

**Table 1:** Effects of *Uvaria comperei* stem crude extract and fractions on albumin denaturation.

Concentration (*μ*g/mL)	Inhibition of albumin denaturation (%)				
—	UCCE	F1	F2	F3	Diclofenac
31.25	29.54	11.63	8.54	30.23	0.00
62.50	47.33	25.58	18.15	38.37	0.00
125.00	79.00	29.07	29.89	47.67	0.00
250.00	100.00	41.86	66.19	56.98	0.00
500.00	100.00	67.44	100.00	60.46	46.26
IC_50_	66.50 ± 0.30^a^	328.00 ± 8.02^c^	193.50 ± 5.65^b^	156.00 ± 7.30^b^	>500^d^

^a,b,c,d^Different letters in the same row indicate a significant difference (*p* < 0.05). UCCE: crude extract of *Uvaria comperei* stems; F1, F2, and F3: fractions from UCCE.

**Table 2:** Effect of *Uvaria comperei* stem crude extract and fractions on haemolytic activity.

Concentration (*μ*g/mL)	Inhibition of heat-induced haemolysis (%)					
—	UCCE	F1	F2	F3	Diclofenac	Ibuprofen
31.25	10.49	40.11	35.63	73.11	26.23	86.28
62.50	25.79	54.32	39.34	79.84	52.35	89.23
125.00	37.59	64.59	38.85	90.00	44.98	89.84
250.00	53.77	65.03	72.68	94.04	69.84	85.08
500.00	75.55	66.34	79.84	95.41	70.71	95.84
IC_50_	219.00 ± 3.46^c^	53.50 ± 1.49^b^	166.00 ± 4.01^c^	<31.25^a^	59.50 ± 2.51^b^	<31.25^a^

^a,b,c^Different letters in the same row indicate a significant difference (*p* < 0.05). UCCE: crude extract of *Uvaria comperei* stems; F1, F2, and F3: fractions of UCCE.

**Table 3:** Effect of fraction F2 on formalin-induced paw licking.

Percentage of inhibition (%)				
Dose (mg/kg)	25	50	100	Indomethacin
First phase	54.38 ± 15.50^a^	60.21 ± 10.20^a^	14.59 ± 6.14^b^	22.91 ± 7.40^b^
Second phase	70.17 ± 7.83^a^	68.67 ± 8.66^a^	38.83 ± 6.34^c^	53.81 ± 10.09^b^

Indomethacin was used at 10 mg/kg. ^a,b,c^Different letters in the same row indicate a significant difference (*p* < 0.05).

**Table 4:** Effect of the fraction F2 on carrageenan-induced hind paw oedema in rats.

Treatment	Dose (mg/kg)	Percentage of inhibition (%)						
		0.5 h	1 h	2 h	3 h	4 h	5 h	6 h
Fraction F2	25	50.00 ± 15.04^a^	77.94 ± 19.43^a^	73.21 ± 4.12^a^	82.50 ± 20.61^a^	88.88 ± 12.83^a^	84.09 ± 15.52^a^	96.50 ± 4.62^a^
	50	38.88 ± 21.27^b^	55.88 ± 26.08^a^	83.33 ± 17.97^a^	93.33 ± 11.54^a^	88.88 ± 19.24^a^	75.75 ± 8.57^a^	87.25 ± 8.26^a^
	100	18.51 ± 8.48^c^	11.76 ± 5.88^c^	28.57 ± 4.35^b^	30.33 ± 1.52^b^	38.88 ± 16.66^b^	72.72 ± 34.81^a^	89.75 ± 2.23^a^

Indomethacin	10	55.55 ± 11.54^a^	35.29 ± 5.80^b^	28.57 ± 4.12^b^	40.00 ± 15.52^b^	22.22 ± 8.23^c^	9.09 ± 4.12^b^	93.75 ± 1.23^a^

^a,b,c^Different letters in the same column indicate a significant difference (*p* ≤ 0.05).

**Table 5:** Effect of F2 fraction administered at 25 mg/kg on serum biochemical parameters.

Group of treatment	Enzymatic activity (IU/L)		
	ALT	AST	*γ*-GT
Control group	74.50 ± 2.12^a^	236.10 ± 16.97^a^	6.05 ± 2.45^a^
Normal group	56.00 ± 2.12^b^	187.30 ± 7.77^b^	4.55 ± 1.98^b^
F2 treatment	44.59 ± 3.53^c^	196.79 ± 20.50^c^	3.50 ± 0.70^b^
Indomethacin group	64.22 ± 4.24^a^	188.87 ± 15.55^c^	1.50 ± 0.70^c^

ALT: alanine aminotransferase; AST: aspartate aminotransferase; *γ*-GT: *γ*-glutamyl transpeptidase. Normal group: rats which did not receive any administration; control group: rats that received the solvent of dissolution of F2. ^a,b,c^Different letters in the same column indicate a significant difference (*p* < 0.05).

**Table 6:** Effect of F2 fraction administered at 25 mg/kg on oxidative stress and acute inflammatory indicators.

Parameters	Oxidative stress		Acute inflammatory
Group of rats	CAT (IU/L)	GSH (*μ*mol/mL)	CRP (mg/mL)
Control group	27.63 ± 0.25^a^	6.73 ± 0.16^b^	n.d.
Normal group	52.91 ± 0.77^b^	13.93 ± 0.16^a^	n.d.
Test group	38.48 ± 0.38^c^	12.37 ± 0.28^a^	n.d.
Indomethacin group	46.27 ± 0.38^b^	7.43 ± 0.41^b^	n.d.

CAT: catalase; GSH: reduced glutathione; CRP: C-reactive protein; n.d.: not detectable (<6 mg/mL). ^a,b,c^Different letters in the same column indicate a significant difference (*p* < 0.05).

**Table 7:** Cytotoxicity of the F2 fraction detected in two cell lines.

	IC_50_ (*μ*g/mL)	
	F2	Podophyllotoxin
Vero cells	82.86 ± 07.13^a^	01.89 ± 0.38^a^
RAW cells	27.73 ± 05.26^b^	0.76 ± 0.09^b^

^a,b^Different letters in the same column indicate a significant difference (*p* < 0.05).

**Table 8:** Compounds identified by HPLC in F2 fraction of *Uvaria comperei* stem extract.

	1	2	3	4	5	6	7	8
F2 fraction			x					x

1, gallic acid; 2, epicatechin; 3, chlorogenic acid; 4, caffeic acid; 5, herniarin; 6, quercetin; 7, imperatorin; 8, catechin.

## Data Availability

The data that support the findings of this study are available from corresponding author upon request.

## Supplementary Materials

Supplementary materials Figures S1 and S2 represent HPLC chromatogram of several standard phenols detected at 340 and 380 nm, respectively. Figures S3 and S4 show the HPLC chromatogram of F2 fraction of *Uvaria comperei* stem extract at 340 and 380 nm, respectively. Figures S5 and S6 display the HPLC chromatogram of F2 fraction combined with the standards at 340 and 380 nm, respectively.
