# Supplementary material for: Anti-inflammatory, Antinociceptive, and Toxicological Properties of Uvaria comperei Stem Crude Extract and Fractions
Source: Biomed Res Int. 2023 Jan 23;2023:2754725. doi: 10.1155/2023/2754725 (PMC9886488; doi:10.1155/2023/2754725)
Supplement: Supplementary materials — Figures S1 and S2 represent HPLC chromatogram of several standard phenols detected at 340 and 380 nm, respectively. Figures S3 and S4 show the HPLC chromatogram of F2 fraction of Uvaria comperei stem extract at 340 and 380 nm, respectively. Figures S5 and S6 display the HPLC chromatogram of F2 fraction combined with the standards at 340 and 380 nm, respectively. [file 2754725.f1.docx]

**Supplementary materials**

S1: HPLC chromatogram of several standard phenols detected at 340 nm.

S2: HPLC chromatogram of several standard phenols detected at 380 nm.

S3: HPLC chromatogram of F2 fraction of *Uvaria comperei* stem extract at 340 nm.

S4: HPLC chromatogram of F2 fraction of *Uvaria comperei* stem extract at 380 nm.

S5: HPLC chromatogram of F2 fraction combined with the standards at 340 nm.

S6: HPLC chromatogram of F2 fraction combined with the standards at 380 nm.
